# Reduced blood-brain barrier penetration of acne vulgaris antibiotic sarecycline compared to minocycline corresponds with lower lipophilicity

**DOI:** 10.3389/fmed.2022.1033980

**Published:** 2022-12-08

**Authors:** Ayman Grada, James Q. Del Rosso, Angela Y. Moore, Linda Stein Gold, Julie Harper, Giovanni Damiani, Katharina Shaw, Sabine Obagi, Raidah J. Salem, S. Ken Tanaka, Christopher G. Bunick

**Affiliations:** ^1^Case Western Reserve University School of Medicine, Cleveland, OH, United States; ^2^JDR Dermatology Research, Las Vegas, NV, United States; ^3^Advanced Dermatology and Cosmetic Surgery, Maitland, FL, United States; ^4^Baylor University Medical Center, Dallas, TX, United States; ^5^Arlington Research Center, Arlington, TX, United States; ^6^Henry Ford Health System, Detroit, MI, United States; ^7^The Dermatology and Skin Care Center of Birmingham, Birmingham, AL, United States; ^8^Clinical Dermatology, Istituto di Ricovero e Cura a Carattere Scientifico (IRCCS) Istituto Ortopedico Galeazzi, Milan, Italy; ^9^Department of Biomedical, Surgical and Dental Sciences, University of Milan, Milan, Italy; ^10^Ph.D. Program in Pharmacological Sciences, Department of Pharmaceutical and Pharmacological Sciences, University of Padua, Padua, Italy; ^11^NYU Langone Health, New York, NY, United States; ^12^USC Neurorestoration Center, Los Angeles, CA, United States; ^13^Medical Affairs, Almirall, LLC, Malvern, PA, United States; ^14^Paratek Pharmaceuticals, Inc., King of Prussia, PA, United States; ^15^Department of Dermatology, Yale School of Medicine, New Haven, CT, United States; ^16^Program in Translational Biomedicine, Yale School of Medicine, New Haven, CT, United States

**Keywords:** acne vulgaris, sarecycline, minocycline, dizziness, blood-brain barrier, lipophilicity, antibiotic

## Abstract

**Background:**

Vestibular side effects such as dizziness and vertigo can be a limitation for some antibiotics commonly used to treat acne, rosacea, and other dermatology indications.

**Objective:**

Unlike minocycline, which is a second-generation tetracycline, sarecycline, a narrow-spectrum third-generation tetracycline-class agent approved to treat acne vulgaris, has demonstrated low rates of vestibular-related adverse events in clinical trials. In this work, we evaluate the brain-penetrative and lipophilic attributes of sarecycline in 2 non-clinical studies and discuss potential associations with vestibular adverse events.

**Methods:**

Rats received either intravenous sarecycline or minocycline (1.0 mg/kg). Blood-brain penetrance was measured at 1, 3, and 6 h postdosing. In another analysis, the lipophilicity of sarecycline, minocycline, and doxycycline was measured *via* octanol/water and chloroform/water distribution coefficients (logD) at pH 3.5, 5.5, and 7.4.

**Results:**

Unlike minocycline, sarecycline was not detected in brain samples postdosing. In the octanol/water solvent system, sarecycline had a numerically lower lipophilicity profile than minocycline and doxycycline at pH 5.5 and 7.4.

**Conclusion:**

The reduced blood-brain penetrance and lipophilicity of sarecycline compared with other tetracyclines may explain low rates of vestibular-related adverse events seen in clinical trials.

## Introduction

Second-generation tetracycline-class antibiotics such as doxycycline and minocycline have been the mainstay treatment of moderate-to-severe acne vulgaris for over 50 years (1–3); however, the use of minocycline is often limited by vestibular side effects such as dizziness and tinnitus, leading to the inclusion of a warning for central nervous system side effects in the minocycline package insert (4). These side effects can impair an individual’s ability to perform daily tasks such as driving (4), thus contributing to the overall burden of managing acne vulgaris. In contrast, vestibular side effects are not typically associated with use of doxycycline (2). The higher lipophilicity of minocycline compared with doxycycline [e.g., distribution coefficient (logD) values of 1.11 (minocycline) vs. 0.95 (doxycycline) (5)] allows for greater penetration of the blood-brain barrier, thereby potentiating vestibular infiltration and by extension, dizziness and vertigo (2).

**FIGURE 1:**
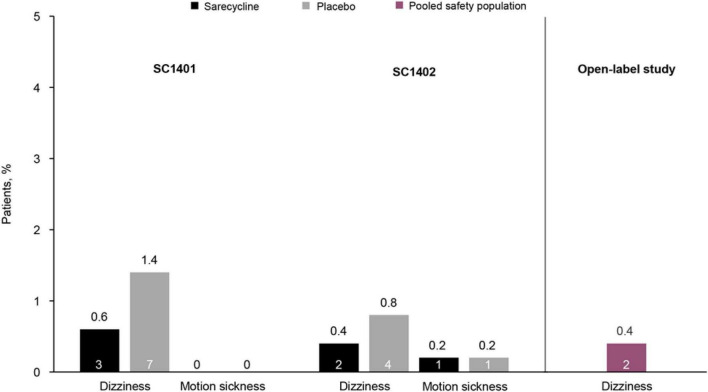
Rates of vestibular-related adverse events in two phase 3 placebo-controlled clinical trials of sarecycline [SC1401 (*N* = 964) and SC1402 (*N* = 1026); representing the safety population] and a phase 3 open-label extension study (*N* = 483) (8, 9). In the open-label study, rates of adverse events were pooled for patients who received placebo in the phase 3 clinical trials and then received sarecycline (*n* = 236) and those who received sarecycline in the phase 3 clinical trials and continued receiving sarecycline in the open-label study (*n* = 247). Motion sickness was not reported in the open-label study. The number of patients who experienced these adverse events is shown in each bar.

Sarecycline is a narrow-spectrum, 3rd generation tetracycline-class oral antibiotic approved by the US Food and Drug Administration (FDA) in 2018 for the treatment of moderate-to-severe acne vulgaris (6, 7). The efficacy and safety of sarecycline have been reported in two phase 3 randomized controlled trials [SC1401 (ClinicalTrials.gov identifier NCT02320149; *N* = 968) and SC1402 (ClinicalTrials.gov identifier NCT02322866); *N* = 1034], and its long-term safety was examined in a 40-week open-label extension study (ClinicalTrials.gov identifier NCT02413346; *N* = 490) (8, 9). Notably, low rates of vestibular-related adverse events (e.g., dizziness, motion sickness) were observed in these studies, and no events of vertigo or tinnitus were reported in patients receiving sarecycline (Figure 1). However, no clinical trials to date have compared sarecycline head-to-head with other tetracycline-class drugs (10), limiting the ability to make direct comparisons for safety and tolerability across therapies. Further, it remains unclear whether the biochemical properties of sarecycline (e.g., blood-brain barrier penetrance, lipophilicity) could be contributing to the low rates of vestibular adverse events in clinical trials.

Here, we investigated the potential relationship between the brain-penetrative and lipophilic attributes of sarecycline from 2 preclinical *in vivo* and *in vitro* analyses. In the first analysis, we examined the ability of sarecycline to penetrate the blood-brain barrier relative to minocycline in a rat model study. In the second analysis, we determined the lipophilicity of sarecycline compared with minocycline and doxycycline.

## Materials and methods

### Blood-brain barrier penetration study

#### Ethics statement

All protocols involving animals were approved by the Institutional Animal Care and Use Committee (IACUC).

### Procedures

Six male Wistar rats (150–200 g; Charles River Laboratories, Wilmington, MA, USA) were used in this study. Each rat was pre-cannulated in the jugular vein. Rats were kept in individual cages, with water and feed *ad libitum*, and alternating 12 h light cycles. Before dosing, rats underwent an overnight fast (∼16 h) in metabolic cages and were weighed to determine dose volume (1.0 ml/kg). The rats were then intravenously (IV) dosed with either sarecycline (*N* = 3) or minocycline (*N* = 3) at a total dose of 1.0 mg/kg, and access to food was restored 2 h after dosing. Subsequently, rats were euthanized *via* CO_2_ and brain samples and whole blood, collected *via* heart puncture, were harvested from 2 rats at each of the following time points: 1, 3, and 6 h postdosing.

### Analysis

The endpoint of this analysis was concentration of sarecycline or minocycline in plasma (μg/ml) and in brain samples (μg/g) at 1, 3, and 6 h postdosing. Plasma and brain homogenate samples were prepared by protein precipitation with acetonitrile, followed by centrifugation. The samples were injected on an API 2000 mass spectrometer (Applied Biosystems, Foster City, CA, USA) and analyzed in positive ion mode using doxycycline as an internal standard. Values were calculated using Analyst 1.2 quantitation software (SciEx, Framingham, MA, USA). Linear through zero regression analysis with no weighting factor was used to determine the calculated concentrations of the injected samples.

### Lipophilicity study

#### Procedure

Sarecycline, minocycline, and doxycycline were each prepared as 1.0 mg/ml stock solutions (4/8 ml) in 3 separate aqueous phase pH buffers (pH 3.5, 5.5, and 7.4). The pH of aqueous phase stock solutions was adjusted to within 0.1 of the desired pH before analysis; stock solutions had a further equilibration period of 2 h before pH adjustment as needed.

Aqueous phase stock solutions were quantified by high performance liquid chromatography (HPLC) before mixing with the organic phase to avoid any potential degradation effects. Next, 2 ml each of aqueous saturated octanol/chloroform and aqueous stock solution were combined and vortex-mixed to encourage interaction between phases. Each experimental condition was conducted in triplicate.

The mixtures were equilibrated at 25°C for 24 h at 750 rotations per minute to allow partitioning. At 2 intervals, the mixtures were vortex-mixed to ensure that an emulsion formed. Following equilibration, the mixtures were allowed to settle before aqueous and organic layers were separated into discrete HPLC vials.

Following 24-h equilibration and phase separation, final pH values of the aqueous phases were recorded. Sarecycline and doxycycline octanol and chloroform phases were diluted as needed with acetonitrile, and minocycline octanol and chloroform phases were diluted as needed with dimethyl sulfoxide. Phases were analyzed by HPLC.

#### Lipophilicity and data analysis

The endpoint of this analysis was the calculation of logD values for each compound at pH 3.5, 5.5, and 7.4. LogD values were calculated by the ratio of the peaks found in the aqueous phase vs. the organic layer, with lower values indicating less lipophilicity and higher values indicating greater lipophilicity. Confirmation of recovery was calculated by determining the concentration of the aqueous stock solution.

## Results

### Blood-brain barrier penetration study

**FIGURE 2:**
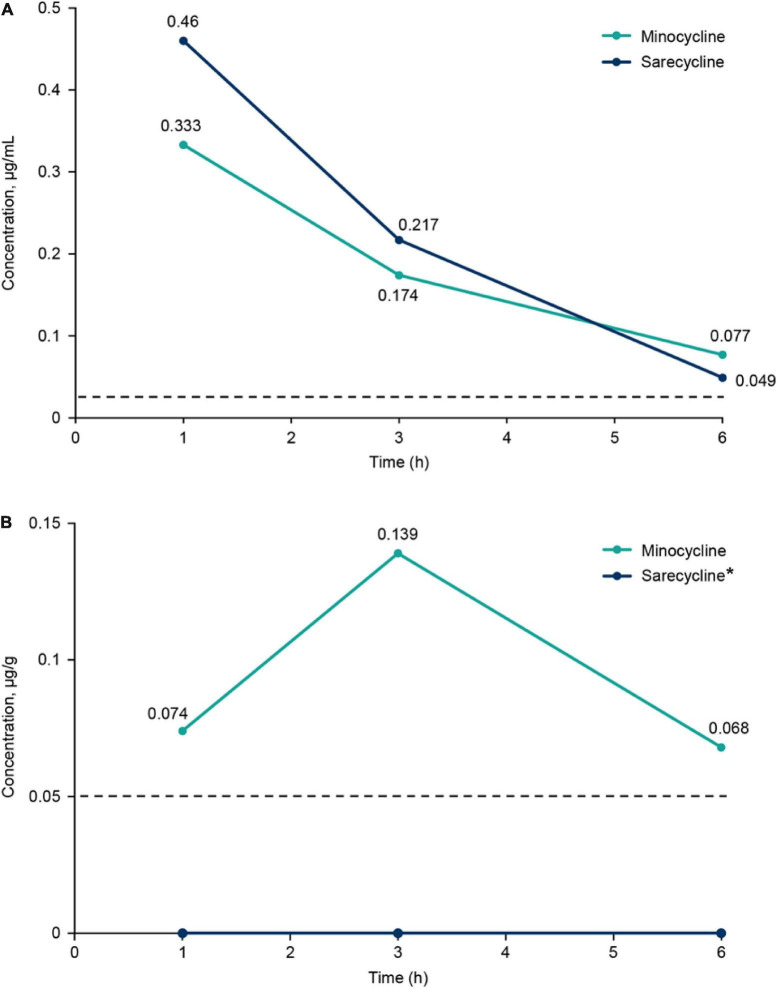
**(A)** Plasma concentrations of sarecycline and minocycline following intravenous administration in rats. **(B)** Brain concentrations of sarecycline and minocycline following intravenous administration in rats. Dashed line indicates limit of quantitation for plasma (0.025 μg/ml) and brain (0.05 μg/g). *Concentration of sarecycline in the brain was below the limit of quantitation at all time points.

Brief results of this study have been reported previously (10). Concentrations of sarecycline and minocycline in rat blood plasma and brain are included in Table 1 and illustrated in Figure 2. Concentrations of sarecycline and minocycline in rat plasma were similar to each other at each measured time point following IV administration [0.460, 0.217, 0.049 ug/ml (sarecycline) versus 0.333, 0.174 and 0.077 ug/ml (minocycline) at hours 1, 3, and 6, respectively]. However, while detectable concentrations of minocycline in the brain were observed at each measured time point (0.074, 0.139, and 0.068 μg/g at hours 1, 3, and 6, respectively), the level of sarecycline remained below the lower limit of quantitation (0.05 μg/g) in all brain samples at each measured time point.

**TABLE 1:** Concentration of minocycline and sarecycline in plasma (μg/ml) and brain (μg/g) after intravenous administration.

Concentration	Time point (hours)		
	1	3	6
**Minocycline**			
Plasma (μg/ml)	0.333	0.174	0.077
Brain (μg/g)	0.074	0.139	0.068
**Sarecycline**			
Plasma (μg/ml)	0.460	0.217	0.049
Brain (μg/g)	BLQ	BLQ	BLQ

LOQ (plasma) = 0.025 μg/ml, LOQ (brain) = 0.05 μg/g. BLQ, below the limit of quantification; LOQ, limit of quantification.

### Lipophilicity study

The logD values of sarecycline, minocycline, and doxycycline in octanol/water and chloroform/water solvent systems at 25°C are reported in Table 2. In the octanol/water system, sarecycline was numerically less lipophilic than minocycline at pH 5.5 and 7.4 (−0.16 vs. 0.09 and −0.26 vs. 0.12, respectively) and numerically more lipophilic at pH 3.5 (−0.30 vs. −1.07, respectively). The lipophilicity of doxycycline was between that of sarecycline and minocycline at pH 5.5 and 7.4.

**TABLE 2:** Distribution coefficients (SD) of sarecycline HCl, doxycycline HCl, and minocycline HCl.

Solvent system	Compound	pH 3.5	pH 5.5	pH 7.4
Octanol/Water at 25°C	Sarecycline HCl	−0.30(0.03)	−0.16(0.01)	−0.26(0.01)
	Doxycycline HCl	−0.01(0.02)	0.00 (0.02)	−0.08(0.03)
	Minocycline HCl	−1.07(0.01)	0.09 (0.02)	0.12 (0.02)
Chloroform/Water at 25°C	Sarecycline HCl	1.14 (0.01)	1.48 (0.01)	1.46 (0.00)
	Doxycycline HCl	−0.15(0.01)	−0.07(0.01)	−0.15(0.01)
	Minocycline HCl	0.07 (0.02)	1.49 (0.02)	1.65 (0.02)

HCl, hydrochloride; SD, standard deviation.

In the chloroform/water system, sarecycline was also numerically less lipophilic than minocycline at pH 7.4, was similarly lipophilic at pH 5.5, and was more lipophilic at pH 3.5. Overall, absolute values of logD were higher in the chloroform/water system than in the octanol/water system for sarecycline (pH 3.5, 1.14 vs. −0.30; pH 5.5, 1.48 vs. −0.16; and pH 7.4, 1.46 vs. −0.26, respectively) and minocycline (pH 3.5, 0.07 vs. −1.07; pH 5.5, 1.49 vs. 0.09; and pH 7.4, 1.65 vs. 0.12, respectively), whereas there was little difference in doxycycline logD values between the 2 solvent systems for each pH solution. Differences in logD values observed between the 2 solvent systems may be attributed to the unique hydrogen bonding capabilities of each system (11).

## Discussion

To explore potential mechanisms of action associated with the development of vestibular adverse events with certain oral tetracyclines, preclinical *in vitro* and *in vivo* analyses were performed to examine the biochemical properties of sarecycline relative to minocycline and doxycycline. In the analysis of *in vivo* blood-brain barrier penetrance, IV administered minocycline was detectable in the brain in rats, whereas sarecycline was not (10). In the analysis of *in vitro* lipophilicity, sarecycline had slightly lower logD values compared with doxycycline and minocycline at pH 5.5 and 7.4 in the octanol/water system. Taken together, these results suggest that the lower lipophilicity and reduced brain penetrance of sarecycline relative to minocycline could explain the lower incidence of vestibular adverse events (dizziness, tinnitus, vertigo) seen in sarecycline clinical trials vs. minocycline clinical trials.

In phase 3 clinical trials of sarecycline, overall rates of vestibular adverse events were low (Figure 1) (8, 9). In two identical, randomized, double-blind, placebo-controlled, phase 3 studies of sarecycline (SC1401 and SC1402), rates of the vestibular-related adverse events dizziness and motion sickness were low (≤ 1%) in 994 patients treated with sarecycline (SC1401 = 481; SC1402 = 513) over 12 weeks (8). Additionally, rates of dizziness were lower in patients receiving sarecycline (0.6 and 0.4%) compared with those receiving placebo (1.4 and 0.8%). In both studies, no events of vertigo or tinnitus were reported in either treatment group. Further, in a phase 3 open-label extension study of 483 patients who completed one of the two phase 3 placebo-controlled 12-week trials, dizziness occurred in 0.4% of patients during up to 40 additional weeks of sarecycline treatment (9). Similarly, no events of vertigo or tinnitus were reported in the extension study. Comparatively, in a placebo-controlled, dose-ranging, 12-week trial of extended-release minocycline in 233 patients with moderate to severe facial acne vulgaris, rates of acute vestibular adverse events (dizziness, vertigo, and ringing in the ears) were most commonly reported during the first 5 days of treatment (12, 13). Incidence of acute vestibular adverse events was dose dependent, occurring in 10.2, 23.7, and 28.3% of patients receiving extended-release minocycline 1, 2, or 3 mg/kg, respectively, versus 16.4% in the placebo group during the first 5 days of treatment. Similarly, in a pooled analysis of phase 3 trials of extended-release 1 mg/kg minocycline, rates of acute vestibular adverse events were 9–10.5% during the first 5 days of treatment (13). In a systematic review representing 226,019 pediatric and adult acne patients, Armstrong et al. reported adverse events associated with sarecycline, minocycline, doxycycline and tetracycline, and showed higher rates of acute vestibular events associated with minocycline (∼10%) (14).

In the current analysis, sarecycline was not detected in rat brain samples up to 6 h following IV dosing (10). In contrast, levels of minocycline were detected in rat brain up to 6 h after IV dosing. A limitation of this animal study is the small sample size, which may make it difficult to generalize. However, the trend seen in the results support previous reports that minocycline has high lipid solubility and, thus, may more readily cross the blood-brain barrier compared with other tetracyclines (15). For instance, a previous report in a canine model indicated that the blood-brain penetrance of minocycline was almost threefold higher than that of doxycycline after IV dosing (16). Although no human studies have definitively confirmed this association, the higher brain penetrance of minocycline is suspected to contribute to its higher rates of associated vestibular-related side effects relative to other tetracyclines (10, 16). Minocycline is considered unacceptable for military aviators and is completely restricted for use because of the risk for central nervous system side effects including vestibular side effects such as light-headedness, dizziness and vertigo (17). Conversely, the low rates of vestibular and phototoxic events seen with narrow-spectrum sarecycline make it an acceptable treatment option for the military population and individuals whose lifestyles and careers would suffer because of vestibular side effects.

Previously published logD values of tetracyclines, especially that of minocycline, are variable and inconsistencies permeate in the dermatology literature. For instance, it was previously reported that minocycline was fourfold more lipophilic than doxycycline and 10-fold more lipophilic than tetracycline at a pH of 5.5 (18, 19). Another analysis found that the logD values for minocycline and doxycycline at pH 5.6 were similar (1.11 and 0.95, respectively) (5). The current analysis indicates that in the octanol/water solvent system at pH 5.5 and 7.4, sarecycline (−0.16 and −0.26, respectively) was slightly less lipophilic than both minocycline (0.09 and 0.12, respectively) and doxycycline (0.00 and −0.08, respectively).

A limitation of this analysis is the interpretation of the lipophilicity results. Because octanol/water solvent systems are the most utilized format for lipophilicity analyses (20), this output was determined to provide the more meaningful result in the current analysis rather than the chloroform/water solvent system. However, caution has been advised for basing pharmacological behavior on partition coefficients, limiting the scope of these results (16). Nevertheless, a strength of the current analysis is that the low lipophilicity of sarecycline is supported by data demonstrating a lack of detectable brain penetrance in rats, which further validates the pharmacological profile of sarecycline.

**FIGURE 3:**
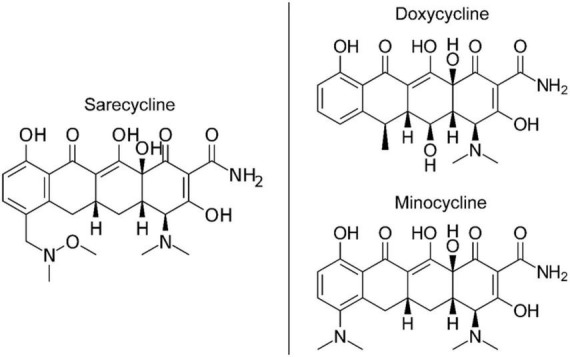
Chemical structures of tetracycline-class antibiotics (26).

The lower lipophilicity and decreased blood-brain barrier penetration observed for sarecycline ultimately must be explained by chemical structure differences between it and minocycline and doxycycline (21–23). While all three drugs share a common naphthacene four-ring core (21, 22), sarecycline is distinguished by a long C7 extension (7-[[methoxy(methyl)amino]methyl) that provides unique and enhanced ribosomal binding through mRNA contact (Figure 3) (21). The C7 moiety contains an oxygen atom (21), which functions as an acceptor of hydrogen bonds, thereby reducing lipophilicity (24, 25). Additional studies are warranted to investigate the mechanism by which sarecycline is associated with lower rates of vestibular adverse events relative to other tetracyclines, but the chemical structure points toward the C7 moiety oxygen. Thus, the new frontier in this work is specific alterations in the chemical properties of dermatologic drugs have potential to make a major impact on reducing real-world adverse events experienced by patients.

While high drug lipophilicity in the skin is a desired attribute as it helps in penetrating and accumulating in the lipid-rich pilosebaceous unit—where acne vulgaris therapeutic target, *Cutibacterium acnes*, resides and proliferates (19)—the increased potential of minocycline to cross the blood-brain barrier compared with other tetracycline-class drugs has served as a purported explanation for the higher rates of vestibular side effects associated with systemic minocycline use in acne treatment, which typically requires a prolonged treatment duration (27–30). Although no confirmatory link has been established, the lack of detectable brain penetrance of sarecycline and its relatively low lipophilicity compared with minocycline as reported in these preclinical studies may correspond with the lower rates of vestibular adverse events observed in clinical trials of sarecycline.

## Data availability statement

The raw data supporting the conclusions of this article will be made available by the authors, without undue reservation.

## Ethics statement

The animal study was reviewed and approved by the Institutional Animal Care and Use Committee (IACUC).

## Author contributions

AG, CB, and ST: conceptualization. AG and ST: methodology. CB: validation. AG: formal analysis. AG and ST: investigation. AG, RS, and ST: resources. AG and LSG: writing—original draft preparation. AG, AM, LSG, CB, JH, GD, KS, RS, and ST: writing—review and editing. CB, SO, and RS: visualization. AG, RS, and ST: supervision. RS: project administration. All authors have read and agreed to the published version of the manuscript.
